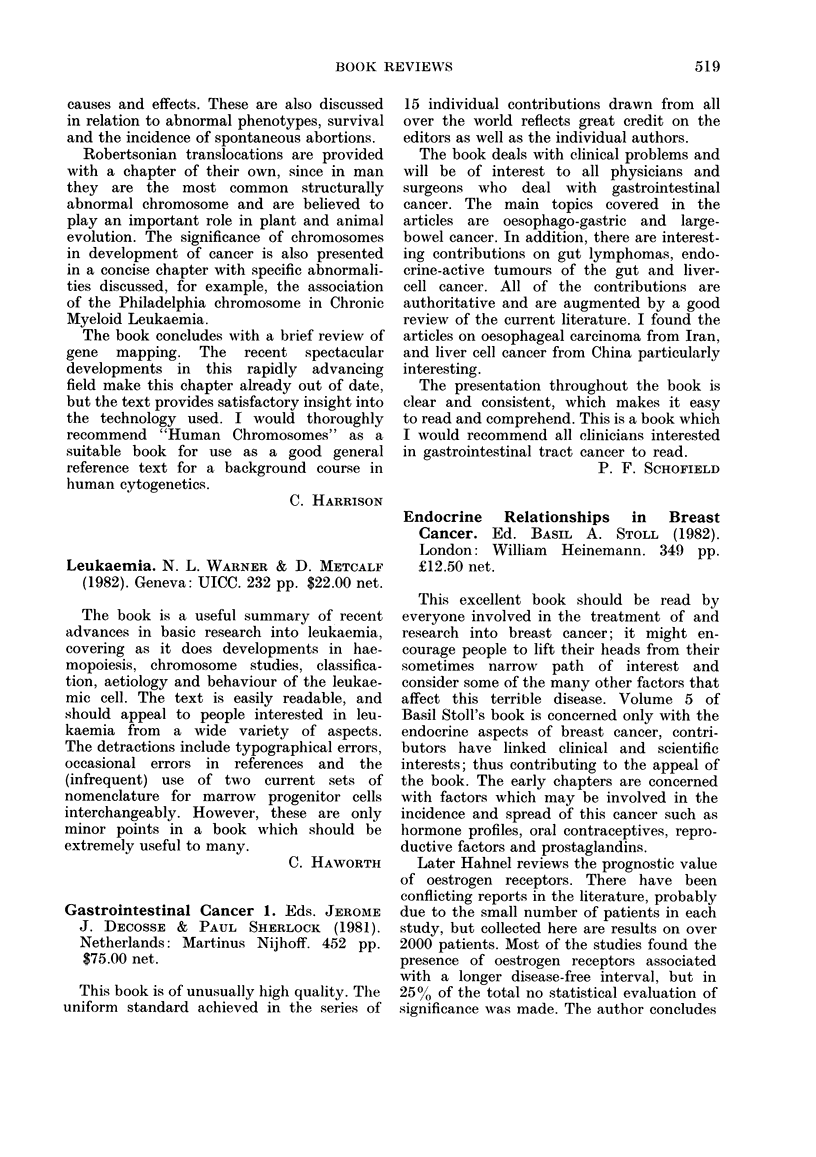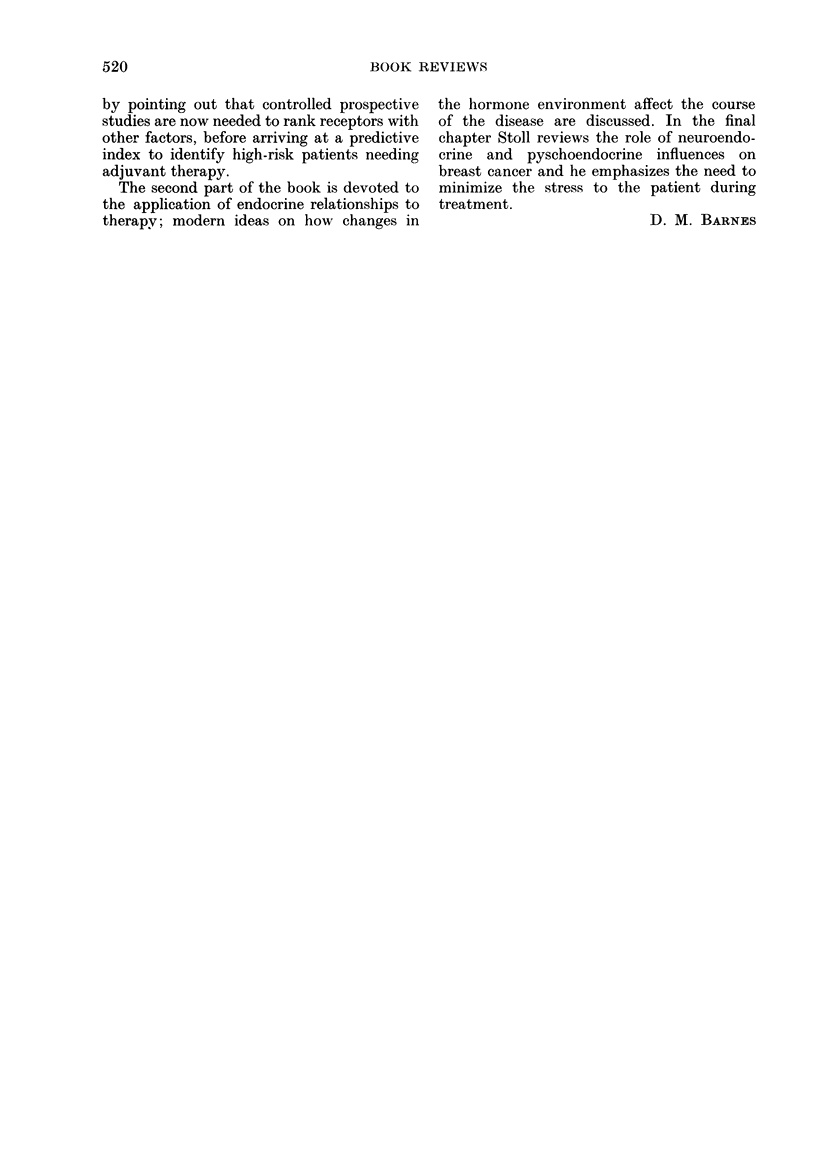# Endocrine Relationships in Breast Cancer

**Published:** 1982-09

**Authors:** D. M. Barnes


**Endocrine Relationships in Breast Cancer.** Ed. BASIL A. STOLL (1982). London: William Heinemann. 349 pp. £12.50 net.

This excellent book should be read by everyone involved in the treatment of and research into breast cancer; it might encourage people to lift their heads from their sometimes narrow path of interest and consider some of the many other factors that affect this terrible disease. Volume 5 of Basil Stoll's book is concerned only with the endocrine aspects of breast cancer, contributors have linked clinical and scientific interests; thus contributing to the appeal of the book. The early chapters are concerned with factors which may be involved in the incidence and spread of this cancer such as hormone profiles, oral contraceptives, reproductive factors and prostaglandins.

Later Hahnel reviews the prognostic value of oestrogen receptors. There have been conflicting reports in the literature, probably due to the small number of patients in each study, but collected here are results on over 2000 patients. Most of the studies found the presence of oestrogen receptors associated with a longer disease-free interval, but in 25% of the total no statistical evaluation of significance was made. The author concludes by pointing out that controlled prospective studies are now needed to rank receptors with other factors, before arriving at a predictive index to identify high-risk patients needing adjuvant therapy.

The second part of the book is devoted to the application of endocrine relationships to therapy; modern ideas on how changes in the hormone environment affect the course of the disease are discussed. In the final chapter Stoll reviews the role of neuroendocrine and pyschoendocrine influences on breast cancer and he emphasizes the need to minimize the stress to the patient during treatment.

D. M. Barnes